# Necroptosis is required for atrial fibrillation and involved in aerobic exercise‐conferred cardioprotection

**DOI:** 10.1111/jcmm.16796

**Published:** 2021-07-20

**Authors:** Yuping Fu, Tiannan Jiang, Hongke Sun, Tong Li, Feng Gao, Boyuan Fan, Xiaoli Li, Xinghua Qin, Qiangsun Zheng

**Affiliations:** ^1^ Department of Cardiology The Second Affiliate Hospital of Xi'an Jiaotong University Xi'an Shaanxi China; ^2^ Department of Internal Medicine Health Care Center Beijing Friendship Hospital Capital Medical University Beijing China; ^3^ School of Life Sciences Northwestern Polytechnical University Xi'an Shaanxi China

**Keywords:** atrial fibrillation, exercise, fibrosis, necroptosis, structural remodeling

## Abstract

Necroptosis, a novel programmed cell death, plays a critical role in the development of fibrosis, yet its role in atrial fibrillation (AF) remains elusive. Mounting evidence demonstrates that aerobic exercise improves AF‐related symptoms and quality of life. Therefore, we explored the role of necroptosis in AF pathogenesis and exercise‐conferred cardioprotection. A mouse AF model was established either by calcium chloride and acetylcholine (CaCl_2_‐Ach) administration for 3 weeks or high‐fat diet (HFD) feeding for 12 weeks, whereas swim training was conducted 60 min/day, for 3‐week duration. AF susceptibility, heart morphology and function and atrial fibrosis were assessed by electrophysiological examinations, echocardiography and Masson's trichrome staining, respectively. Both CaCl_2_‐Ach administration and HFD feeding significantly enhanced AF susceptibility (including frequency and duration of episodes), left atrial enlargement and fibrosis. Moreover, protein levels of necroptotic signaling (receptor‐interacting protein kinase 1, receptor‐interacting protein kinase 3, mixed lineage kinase domain‐like protein and calcium/calmodulin‐dependent protein kinase II or their phosphorylated forms) were markedly elevated in the atria of AF mice. However, inhibiting necroptosis with necrostatin‐1 partly attenuated CaCl_2_‐Ach (or HFD)‐induced fibrosis and AF susceptibility, implicating necroptosis as contributing to AF pathogenesis. Finally, we found 3‐week swim training inhibited necroptotic signaling, consequently decreasing CaCl_2_‐Ach‐induced AF susceptibility and atrial structural remodeling. Our findings identify necroptosis as a novel mechanism in AF pathogenesis and highlight that aerobic exercise may confer benefits on AF via inhibiting cardiac necroptosis.

## 1 INTRODUCTION

Atrial fibrillation (AF), a highly prevalent arrhythmia in clinical practice associated with increased morbidity and mortality, has become a public health issue.^1^ The underlying mechanism of AF pathogenesis is widely explored, and structural remodeling, consisting of elevated atrial myocyte death and resultant fibrosis, as well as compensatory atrial dilation, is thought to be pivotal in this process.^2^ Apoptosis and necrosis, two major forms of cell death, are crucial pathological basis of cardiovascular diseases.^3^ The role of apoptosis in AF has been intensively explored,^4^ ^,^ ^5^ yet whether necroptosis, a recently defined programmed necrosis, contributes to AF remains elusive.

Necroptosis, combining features of both necrosis and apoptosis, is a newly caspase‐independent programmed form of necrosis.^6^ It is now attracting more and more attention that necroptosis contributes to the development of various cardiovascular diseases,^7^ and especially acts as a molecular target for age‐related heart disease.^8^ Necroptosis is mediated by a signaling complex called “necrosome,” containing the receptor‐interacting protein kinase 1—the receptor‐interacting protein kinase 3 (RIP1‐RIP3) complex and its downstream substrates—the mixed lineage kinase domain‐like protein (MLKL),^9^ or calcium/calmodulin‐dependent protein kinase II (CaMKII),^10^ which ultimately leading to cell membrane rupture and cell death. Increasing evidence demonstrates inhibition of necroptotic signaling with either genetic manipulation (RIP3 knockout mice) or pharmacological drugs (necrostatin‐1, Nec‐1) protect against fibrosis,^11^, ^12^ the hallmark of atrial structural remodeling of AF. In line with this, silencing its downstream substrate CaMKII or inhibiting its activity by KN93 is also implicated to reduce transforming growth factor‐β (TGF‐β)‐mediated fibrosis.^13^ Therefore, necrotic loss of cardiomyocytes by necroptosis might lead to cardiac fibrosis and extracellular matrix remodeling,^2^, ^14^ thereby providing a structural substrate for AF.

Physical exercise is a non‐invasive therapeutic approach for prevention and treatment of cardiovascular diseases, such as coronary artery disease, hypertension and diabetes, described as the notion “exercise is medicine.”^15^ However, in the aspect of AF, the effects of exercise are more complicated and depend on exercise frequency, intensity, time and type (the FITT principle).^16^ Both clinical and experimental studies demonstrated high‐intensity exercise might raise the risk of AF and are even used as a means to establish an AF model in animal studies.^17^, ^18^, ^19^ However, lately, emerging clinical evidence supported low to moderate regular exercise exerted beneficial effects against AF,^20^ probably via reversing atrial remodeling, improving risk factors, improving autonomic tone and reducing inflammation.^16^ Mozaffarian et al.^21^ reported moderate‐intensity aerobic exercise reduced AF risk in older adults. Hegbom et al.^22^ demonstrated short‐term exercise improved AF‐related symptoms and quality of life in patients with chronic AF. However, most of these studies are population‐based studies, and rare animal studies are conducted and the underlying mechanism needs further exploration.

A recent experimental research has revealed high‐intensity interval training could reduce myocardial infarction area and cardiac remodeling by targeting necroptosis in a rat myocardial ischemia/reperfusion model,^23^ suggesting the involvement of necroptosis in exercise‐conferred cardioprotection. Thus, in the present study, using a mouse model of calcium chloride and acetylcholine (CaCl_2_‐Ach) (or high‐fat diet, HFD)‐induced AF and swim training, we tentatively explored (1) the role of necroptosis in AF pathogenesis and (2) whether swim training could attenuate AF and the role of necroptosis in this process.

## 2 MATERIEL AND METHODS

### 2.1 Animal models

Male C57BL/6J mice aged 8–10 weeks were purchased from the Experimental Animal Signaling of Xi'an Jiaotong University (Xi'an, China). The animal experiments were performed in accordance with the Guide for the Care and Use of Laboratory Animals published by the US National Institutes of Health (Publication No. 85–23, revised 1996) and approved by the Institutional Animal Ethics Committee of Xi'an Jiaotong University.

Establishment of CaCl_2_‐Ach (or HFD)‐induced AF model and swim training protocol was described below. After AF model establishment concurrently with pharmacological or physical intervention, echocardiography and AF induction by burst pacing were performed before tissue sampling. There were no unexpected animal deaths during the experimental procedures in this study. Euthanasia was performed by heart excision in deep anesthesia, and atria samples were either fixed in 4% paraformaldehyde for histological experiments or snap‐frozen in liquid nitrogen and stored at −80℃ until analysis. All animal procedures were performed under anesthesia either by sodium pentobarbital (60 mg/kg, intraperitoneal) injection or isoflurane (1.5%) inhalation, unless otherwise specified. Detailed experimental framework was shown in Figure S1.

#### 2.1.1 Mouse model of CaCl_2_‐Ach‐induced AF

A mouse AF model was performed via tail vein drug injection following Chen et al.^24^ with slight modifications. Briefly, intravenous injection of CaCl_2_ (10 mg/kg)‐Ach (66 µg/kg) mixture lasted for 3 weeks when AF frequency and duration in CaCl_2_‐Ach group were significantly increased compared with Saline group (Figure S2).

#### 2.1.2 Mouse model of HFD‐induced AF

Mice were fed HFD (Research Diets, D12492, Protein 20 kcal%, Carbohydrate 20 kcal%, Fat 60 kcal%, total energy 5.24 kcal/gm) for 12 weeks to establish an obesity‐induced AF model. A normal‐fat diet (NFD) (Protein 20 kcal%, Carbohydrate 70 kcal%, Fat 10 kcal%, total energy 3.84 kcal/gm) was provided for NFD group.

#### 2.1.3 Swim training protocol

Mice were pre‐adapted to swim training with a 10‐min session for the first day and then progressively increased to 60 min daily in a week and swam 60 min/day for 3 weeks.

### 2.2 Non‐invasive transthoracic echocardiography

After skin preparation of the chest and upper abdomen, mice were fixed on the operating table in supine position and anaesthetized with 1.5% isoflurane. Non‐invasive transthoracic echocardiography was carried out to assess heart morphology and function using the Vevo2100 Imaging system (VisualSonics, Toronto, Ontario, Canada) equipped with a 30 MHz phased‐array transducer. Left atrium (LA) diameter was determined from M‐mode at end‐systole. All these images were processed using Vevolab 3.1 software (VisualSonics).

### 2.3 AF induction by burst pacing

Atrial fibrillation induction by high‐frequency burst pacing was performed with a programmable electrical stimulator (VCS3001, MappingLab Ltd., UK) according to a protocol described by Fan et al.^25^ with slight modifications. Briefly, anaesthetized mice were fixed on the operating table in supine position with continuous surface lead electrocardiogram (ECG) recorded via a BL‐420S biological signal collection system (Chengdu Taimeng Technology Co., Ltd., Chengdu, China). A 30‐Hz burst pacing (pulse width 1 ms, 2× threshold current) on the high right atrium was performed to induce AF after thoracotomy with atrial ECG recorded. AF was successfully induced when P wave disappeared in combination with irregular RR intervals on surface ECG, and rapid and irregular atrial waves appeared on atrial ECG, lasting for at least 1 s. AF frequency (number of incidences per animal) and AF duration (in seconds) were used to evaluate AF susceptibility.

### 2.4 Histological evaluation of atrial fibrosis

After fixation in 4% paraformaldehyde overnight, atria were embedded in paraffin and sliced into 5‐µm‐thick sections. Then, atria sections were stained with Masson trichrome to evaluate the degree of atrial fibrosis and images were captured under the fixed microscope illumination settings (Olympus, Tokyo, Japan). Fibrosis was analyzed and quantified using ImageJ software (National Institutes of Health, Bethesda, Maryland, USA). Degree of fibrosis was evaluated and calculated by measuring the blue regions (collagen) relative to total tissue area and represented the average of three randomly selected fields (400× magnification) per slide.

### 2.5 Protein extraction and western blot analysis

Western blot was performed according to standard protocols. Atrial tissues were homogenized, and proteins were isolated using radio‐immunoprecipitation assay lysis buffer containing protease inhibitor cocktail (Roche, Mannheim, Germany). Protein concentration was determined using a BCA assay kit (TDY Biotech, Beijing, China). Proteins were separated by 10% SDS‐polyacrylamide gel electrophoresis and transferred to polyvinylidene fluoride membranes. Subsequently, the membranes were blocked with 5% skim milk for 1 h at room temperature. Diluted primary antibodies against RIP1 (1:1000, Cell Signaling Technology, Danvers, MA, USA), p‐RIP1 (1:1000, Cell Signaling Technology), RIP3 (1:1000, Cell Signaling Technology), p‐RIP3 (1:1000, Cell Signaling Technology), CaMKII (1:1000, Cell Signaling Technology), p‐CaMKII (1:1000, Cell Signaling Technology), MLKL (1:2000, Millipore, Merck KGaA, Darmstadt, Germany), AMPK (1:1000, Cell Signaling Technology), p‐AMPK (1:1000, Cell Signaling Technology), mTOR (1:1000, Cell Signaling Technology), p‐mTOR (1:1000, Cell Signaling Technology), Beclin1 (1:1000, Cell Signaling Technology), LC3 (1:2000, Novus Biologicals, Littleton, USA), p62/SQSTM1 (1:10,000, Abcam, Cambridge, MA, USA) and GAPDH (1:1000, CoWin Biosciences, Beijing, China) were incubated overnight at 4℃ followed by the corresponding secondary antibodies (1:5000, TDY Biotech) incubated at room temperature. Band signals were detected with Tanon‐5200 Multi chemiluminescent imaging system (Tanon, Shanghai, China) and quantified by densitometry (standardized to the GAPDH band) using Quantity One software (Bio‐Rad, Hercules, California, USA).

### 2.6 Immunofluorescence staining

Four percent paraformaldehyde‐fixed atria were embedded in OCT compound, snap‐frozen and sliced into 5‐µm‐thick sections using a freezing microtome (Leica, Wetzlar, Germany). Cryosections were fixed and permeabilized with ice‐cold acetone and blocked with 5% BSA at room temperature before incubated with mouse anti‐MLKL antibody (1:200, Millipore, Merck KGaA) at 4℃ overnight. FITC‐labeled goat‐anti‐mouse IgG (1:200, Beyotime, Shanghai, China) was added sequentially, and the nuclei were stained with DAPI. Finally, the slides were mounted with 50% glycerol and observed under confocal microscope (Zeiss, Oberkochen, Germany) for fluorescent signal analysis.

### 2.7 Statistical analysis

All values are presented as mean ±SEM and analyzed by SPSS version 22.0 (IBM, Cologne, Germany) or GraphPad Prism software version 8.0 (GraphPad, La Jolla, CA). Student's *t* test or one‐way ANOVA with Bonferroni's post hoc multiple comparison test was performed for comparison among treatment groups. *p* < 0.05 was considered to be statistically significant.

## 3 RESULTS

### 3.1 Inhibiting necroptosis decreased CaCl_2_‐Ach‐induced AF susceptibility and reversed atrial structural remodeling

**FIGURE 1:**
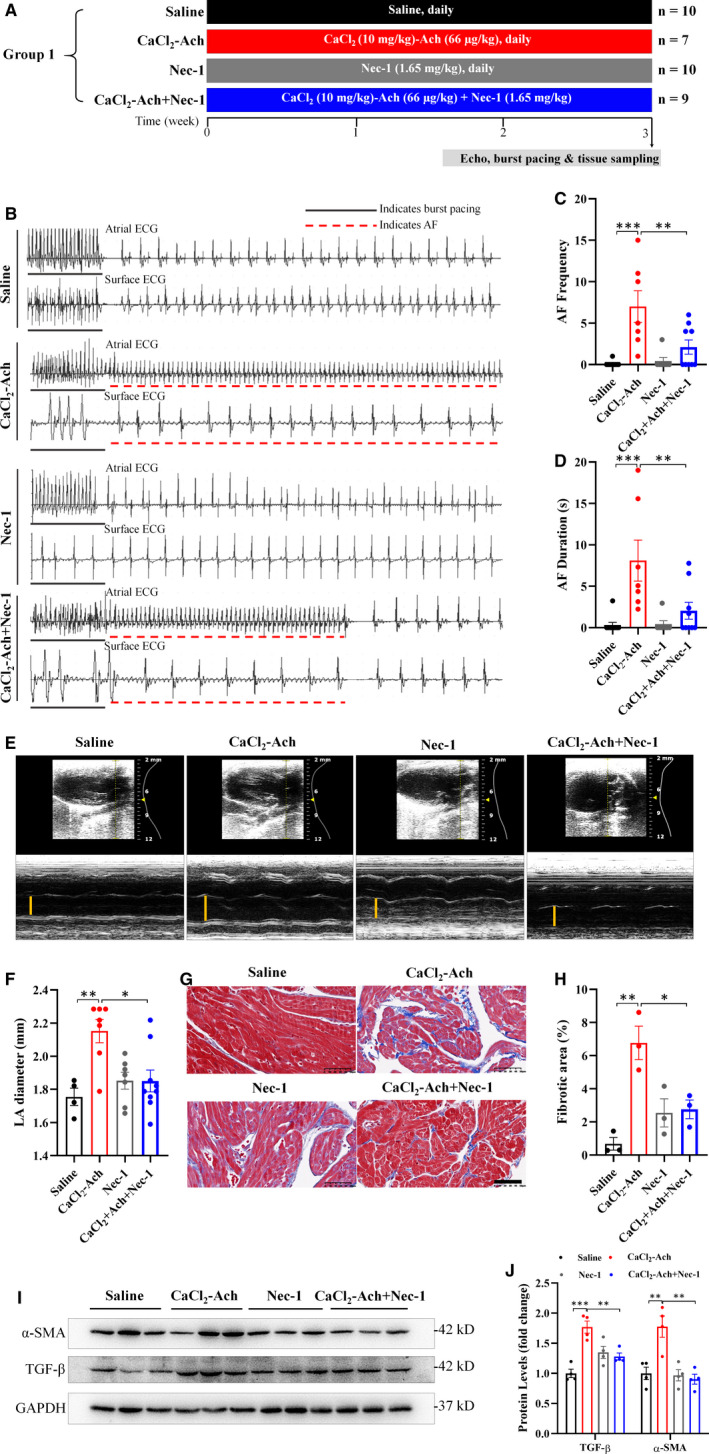
Three‐week CaCl_2_‐Ach administration promoted AF susceptibility and atrial structural remodelling while inhibiting necroptosis reversed these effects. (A) Experimental protocols of Group 1. (B)–(D) Representative ECGs and bar graphs showed burst pacing‐induced AF with increased AF frequency and duration in CaCl_2_‐Ach group (*n* = 7) compared with Saline group (*n* = 10), and less episodes of AF with decreased AF frequency and duration in CaCl_2_‐Ach+Nec‐1 group (*n* = 9) compared with CaCl_2_‐Ach group. (E)–(F) Representative echocardiography and bar graph showed significantly increased LA diameter in CaCl_2_‐Ach group (*n* = 7) compared with Saline group (*n* = 4) and decreased LA diameter in CaCl_2_‐Ach+Nec‐1 group (*n* = 9) compared with CaCl_2_‐Ach group. (G)–(H) Representative Masson's trichrome staining and quantitative analysis of fibrotic area exhibited more collagen deposition (blue) in CaCl_2_‐Ach group (*n* = 3) under 400× magniﬁcation compared with Saline group (*n* = 3) and less fibrosis in CaCl_2_‐Ach+Nec‐1 group (*n* = 3) compared with CaCl_2_‐Ach group. (I)–(J) Representative images and quantitative analysis showed elevated levels of fibrosis‐related gene: α‐SMA and TGF‐β in CaCl_2_‐Ach group compared with Saline group but reversed effect after Nec‐1 administration. Scale bar: 50 µm. ^*^ *p* < 0.05, ^**^ *p* < 0.01, ^***^ *p* < 0.001. Ach, acetylcholine; AF, atrial fibrillation; CaCl_2_, calcium chloride; ECG, electrocardiogram; LA, left atrium; Nec‐1, necrostatin‐1

As calcium overload and Ach releasing are contributing factors for triggering AF, we established a mouse AF model by intravenous administration of CaCl_2_‐Ach mixture daily and used a special necroptosis inhibitor, Nec‐1 (1.65 mg/kg), to inhibit necroptosis to identify the role of necroptosis in AF pathogenesis. Mice were randomized into 4 groups: Saline group (*n* = 10), CaCl_2_‐Ach group (*n* = 7), Nec‐1 group (*n* = 10) and CaCl_2_‐Ach+Nec‐1 group (*n* = 9) (Figure 1A). To determine the optimal time of CaCl_2_‐Ach administration, high‐frequency atrial burst pacing was applied to assess AF susceptibility every week, and the results demonstrated that AF frequency and duration were significantly higher in CaCl_2_‐Ach group compared with Saline group only after 3 weeks of CaCl_2_‐Ach administration, whereas no significant changes were observed between groups earlier (Figures 1B–D and S2). Moreover, LA enlargement (1.755 ± 0.052 mm vs. 2.152 ± 0.070 mm, Table S1 and Figure 1E,F), a strong risk factor of AF, and fibrosis, another core process involved in atrial structural remodeling in AF pathogenesis, were significantly enhanced after 3 weeks of CaCl_2_‐Ach administration (Figure 1G–J). Overall, electrophysiological and echocardiographic results suggested a success in CaCl_2_‐Ach‐induced AF model establishment.

**FIGURE 2:**
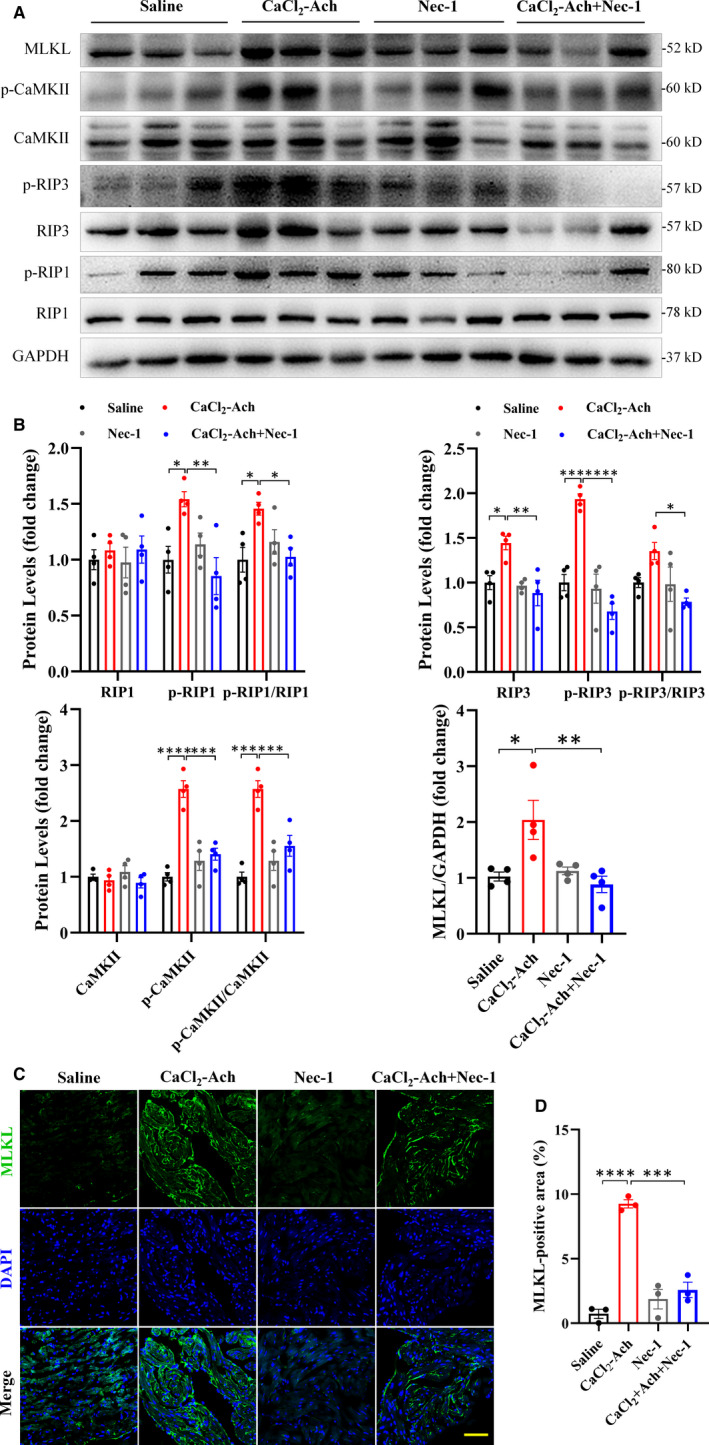
Cardiac necroptosis occurred in CaCl_2_‐Ach‐induced AF mice while Nec‐1 administration inhibiting this effect. (A)–(B) Representative images and quantitative analysis showed significantly elevated RIP1, RIP3, CaMKII or their phosphorylated levels, as well as expression of MLKL in CaCl_2_‐Ach group (*n* = 4) compared with Saline group (*n* = 4) and inhibited necroptotic signalling in CaCl_2_‐Ach+Nec‐1 group (*n* = 4) compared with CaCl_2_‐Ach group. (C)–(D) Representative images and quantitative analysis showed significantly increased cell membrane translocation of MLKL in CaCl_2_‐Ach group (*n* = 3) compared with Saline group (*n* = 3) and attenuating effect in CaCl_2_‐Ach+Nec‐1 group (*n* = 3) compared with CaCl_2_‐Ach group. Scale bar: 100 µm. ^*^ *p* < 0.05, ^**^ *p* < 0.01, ^***^ *p* < 0.001, ^****^ *p* < 0.0001. Ach, acetylcholine; Ach, acetylcholine; AF, atrial fibrillation; CaCl_2_, calcium chloride; Nec‐1, necrostatin‐1

Next, we explored the level of cardiac necroptosis in CaCl_2_‐Ach‐induced AF mice and found protein levels of necroptotic signaling (RIP1, RIP3, CaMKII and MLKL or their phosphorylated forms) (Figure 2A,B), as well as cell membrane translocation of MLKL (Figure 2C,D), were significantly increased. Inhibiting necroptosis with Nec‐1 (Figure 2) apparently attenuated CaCl_2_‐Ach‐induced AF susceptibility as evidenced by decreased AF frequency and duration (Figure 1B–D). We also found atrial structural remodeling reversed as indicated by decreased LA diameter and atrial fibrosis after 3‐week Nec‐1 administration (Figure 1E–J).

Taken together, decreased AF susceptibility and reversed atrial structural remodeling in AF mice after pharmacological inhibition of necroptosis indicated a critical role of necroptosis in AF pathogenesis.

### 3.2 Inhibiting necroptosis decreased HFD‐induced AF susceptibility and mitigated atrial fibrosis

**FIGURE 3:**
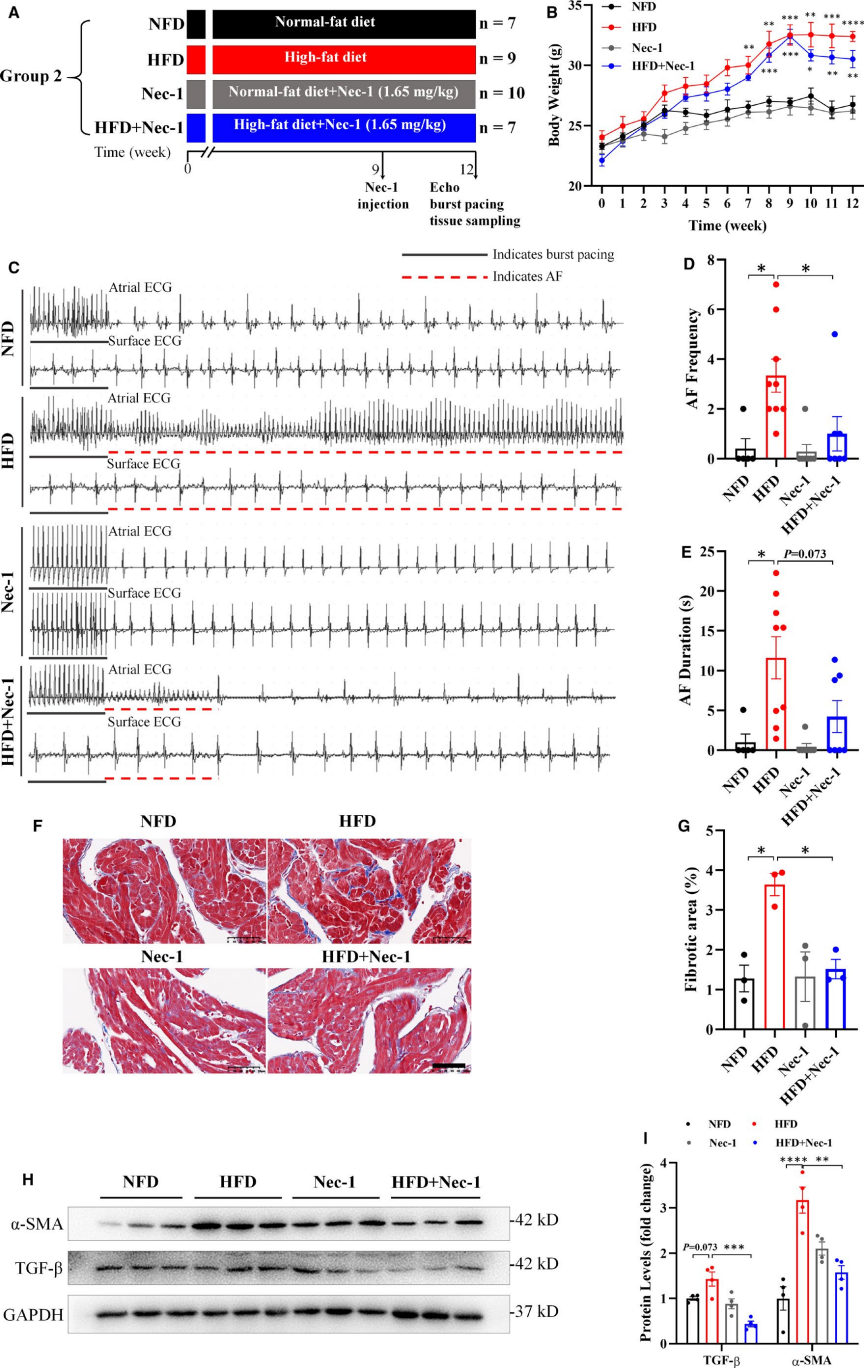
Twelve‐week HFD feeding promoted AF susceptibility and atrial fibrosis while inhibiting necroptosis reversed these effects. (A) Experimental protocols of Group 2. (B) Body weight apparently increased in both HFD‐fed groups compared with NFD group or Nec‐1 group at the end of week 8 and met the standard of obesity at the end of week 9. (C)–(E) Representative ECGs and bar graphs showed burst pacing‐induced AF with increased AF frequency and duration in HFD group (*n* = 9) compared with NFD group (*n* = 5), and less episodes of AF with decreased AF frequency and duration in HFD+Nec‐1 group (*n* = 7) compared with HFD group. (F)–(G) Representative Masson's trichrome staining and quantitative analysis of fibrotic area exhibited more collagen deposition (blue) in HFD group (*n* = 3) under 400× magniﬁcation compared with NFD group (*n* = 3) and less fibrosis in HFD+Nec‐1 group (*n* = 3) compared with HFD group. (H)–(I) Representative images and quantitative analysis showed elevated levels of fibrosis‐related gene: α‐SMA and TGF‐β in HFD group compared with NFD group but reversed effect after Nec‐1 administration. Scale bar: 50 µm. ^*^ *p* < 0.05, ^**^ *p* < 0.01, ^***^ *p* < 0.001, ^****^ *p* < 0.0001. AF, atrial fibrillation; ECG, electrocardiogram; HFD, high‐fat diet; Nec‐1, necrostatin‐1; NFD, normal‐fat diet

To further investigate the role of necroptosis in different AF models, we established a HFD‐induced mouse AF model by feeding HFD for 12 weeks and inhibited necroptosis via Nec‐1 injection in the last 3 weeks from the tail vein. Mice were randomly divided into 4 groups: NFD group (*n* = 7), HFD group (*n* = 9), Nec‐1 group (*n* = 10) and HFD+Nec‐1 group (*n* = 7) (Figure 3A). HFD‐fed mice started to meet the standard of obesity at the end of week 9 (body weight increased by 20% compared with NFD group, Figure 3B) and showed apparently enhanced AF susceptibility at the end of week 12 (Figure 3C–E and S3). Compared with NFD group, HFD group showed significantly increased AF frequency and duration after burst pacing (Figure 3C–E) and apparent atrial fibrosis (Figure 3F–I), suggesting a success in HFD‐induced AF model establishment.

**FIGURE 4:**
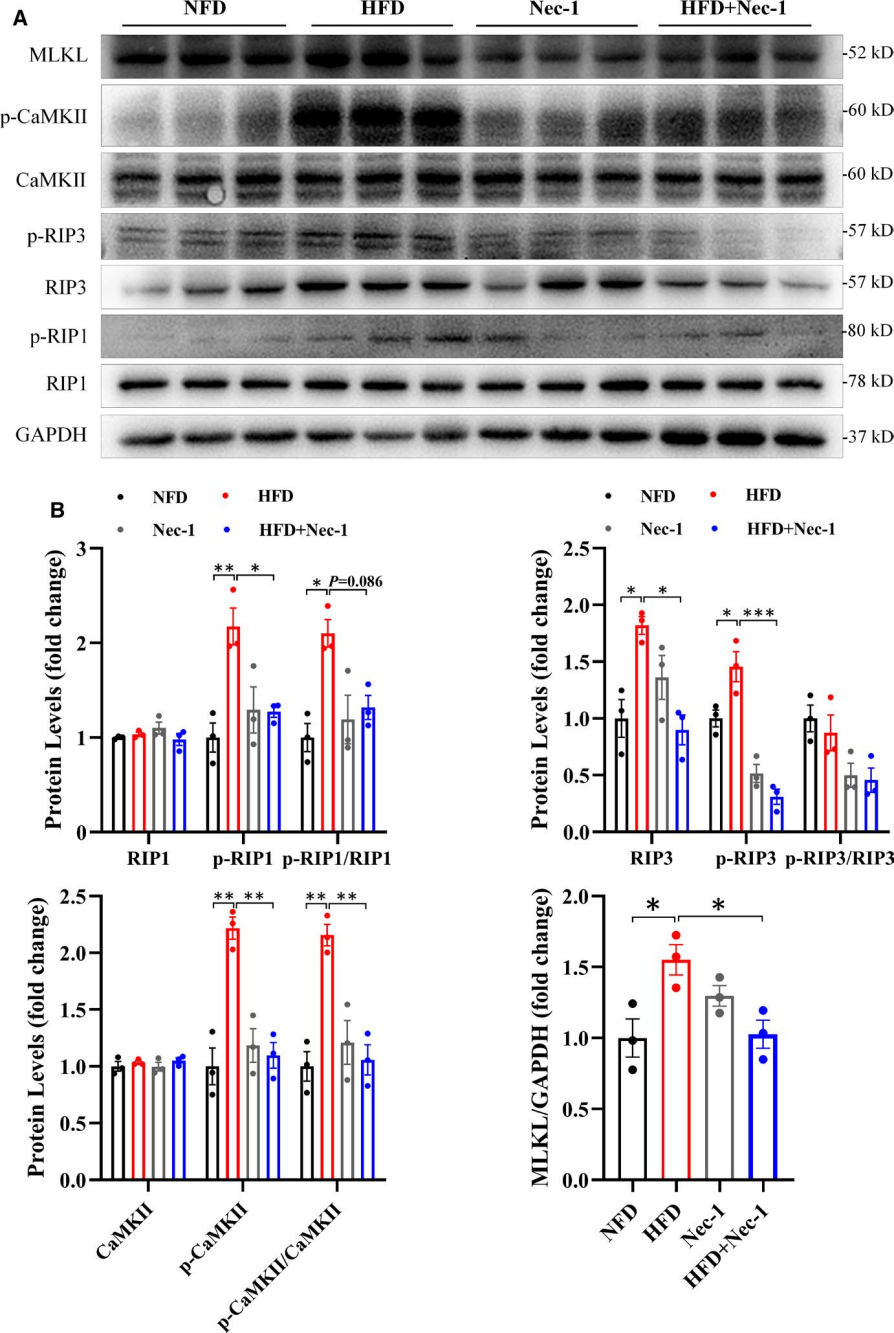
Cardiac necroptosis occurred in HFD‐induced AF mice while Nec‐1 administration inhibiting this effect. (A)–(B) Representative images and quantitative analysis showed significantly elevated RIP1, RIP3, CaMKII and MLKL or their phosphorylated levels in HFD group (*n* = 3) compared with NFD group (*n* = 3) and inhibited necroptotic signalling in HFD+Nec‐1 group (*n* = 3) compared with HFD group. ^*^ *p* < 0.05, ^**^ *p* < 0.01, ^***^ *p* < 0.001. AF, atrial fibrillation; HFD, high‐fat diet; Nec‐1, necrostatin‐1; NFD, normal‐fat diet

Furthermore, cardiac necroptosis occurred in HFD‐induced AF mice as evidenced by increased protein levels of RIP1, RIP3, CaMKII and MLKL or their phosphorylated forms (Figure 4). However, inhibiting necroptosis with Nec‐1 (Figure 4) decreased HFD‐induced AF susceptibility and mitigated atrial fibrosis (Figure 3C–I), indicating a key role of necroptosis in AF pathogenesis in HFD‐induced AF mice, consistent with the results in CaCl_2_‐Ach‐induced AF model.

### 3.3 Swim exercise training decreased CaCl_2_‐Ach‐induced AF susceptibility and reversed atrial structural remodeling

**FIGURE 5:**
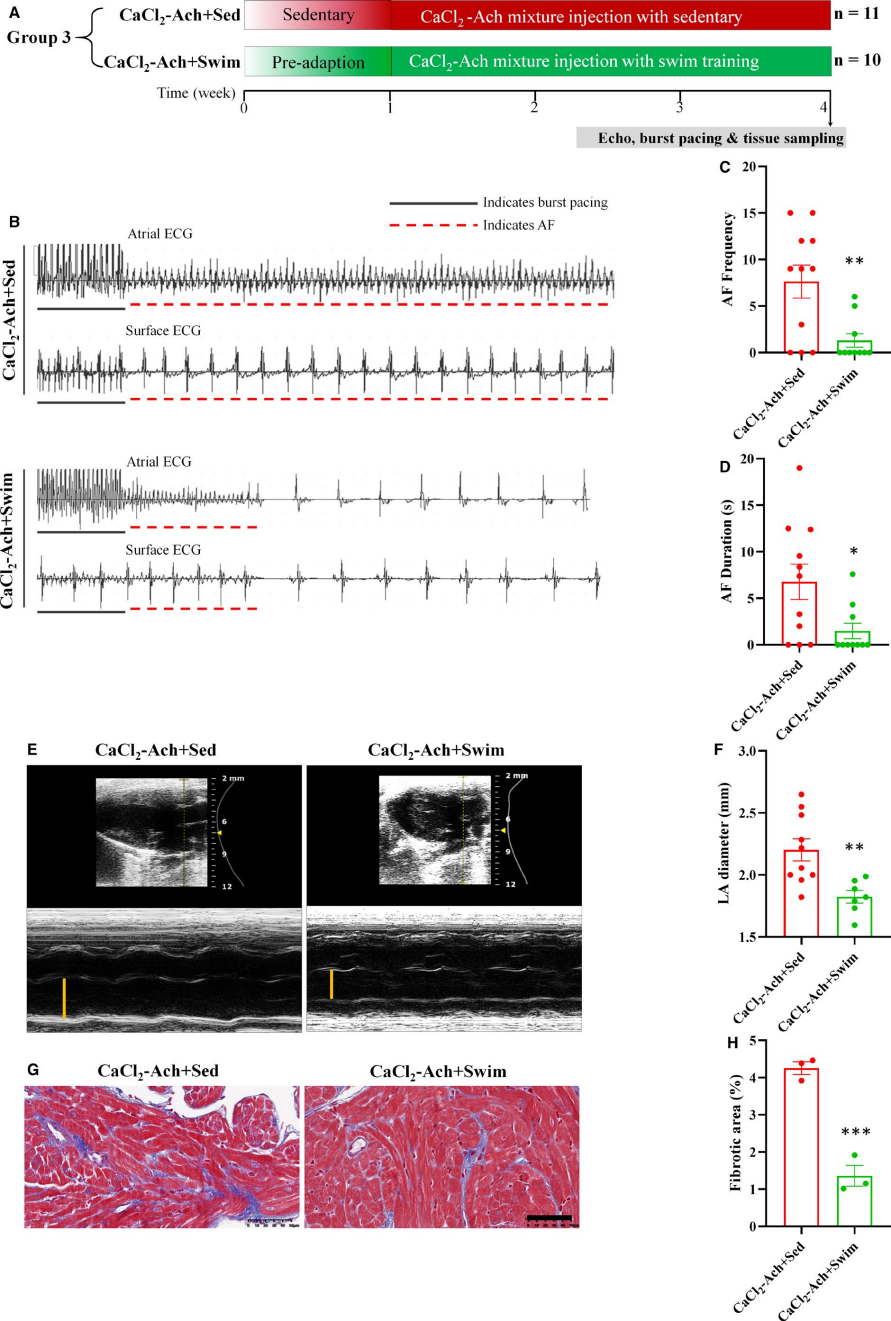
Swim exercise training decreased CaCl_2_‐Ach‐induced AF susceptibility and reversed atrial structural remodelling. (A) Experimental protocols of Group 3. (B)–(D) Representative ECGs and bar graphs showed decreased AF frequency and duration in CaCl_2_‐Ach+Swim group (*n* = 10) compared with CaCl_2_‐Ach group (*n* = 11). (E)–(F) Representative echocardiography and bar graph showed significantly decreased LA diameter in CaCl_2_‐Ach+Swim group (*n* = 7) compared with CaCl_2_‐Ach group (*n* = 10). (G)–(H) Representative Masson's trichrome staining and quantitative analysis showed significantly decreased fibrosis in CaCl_2_‐Ach+Swim group (*n* = 3) compared with CaCl_2_‐Ach group (*n* = 3). Scale bar: 50 µm. ^*^ *p* < 0.05, ^**^ *p* < 0.01, ^***^ *p* < 0.001. Ach, acetylcholine; AF, atrial fibrillation; CaCl2, calcium chloride; ECG, electrocardiogram; LA, left atrium; Sed, sedentary

To identify whether swim exercise training could attenuate AF, mice were randomized into 2 groups: CaCl_2_‐Ach+sedentary (Sed) group (*n* = 11) and CaCl_2_‐Ach+Swim group (*n* = 10) (Figure 5A), and AF susceptibility and atrial structural remodeling were assessed in both groups. The electrophysiological results showed swim training attenuated CaCl_2_‐Ach‐induced AF susceptibility as evidenced by decreased AF frequency and shorter duration (Figure 5B–D). Moreover, echocardiography revealed that swim training significantly decreased LA diameter in CaCl_2_‐Ach‐induced AF mice (Table S1 and Figure 5E,F). In line with this finding, Masson's trichrome staining showed that the degree of fibrosis was significantly lower in CaCl_2_‐Ach+Swim group compared with CaCl_2_‐Ach+Sed group (Figure 5G,H). All the findings above demonstrated that swim exercise training could apparently attenuate CaCl_2_‐Ach‐induced AF.

### 3.4 Swim exercise training inhibited cardiac necroptosis in CaCl_2_‐Ach‐induced AF mice

**FIGURE 6:**
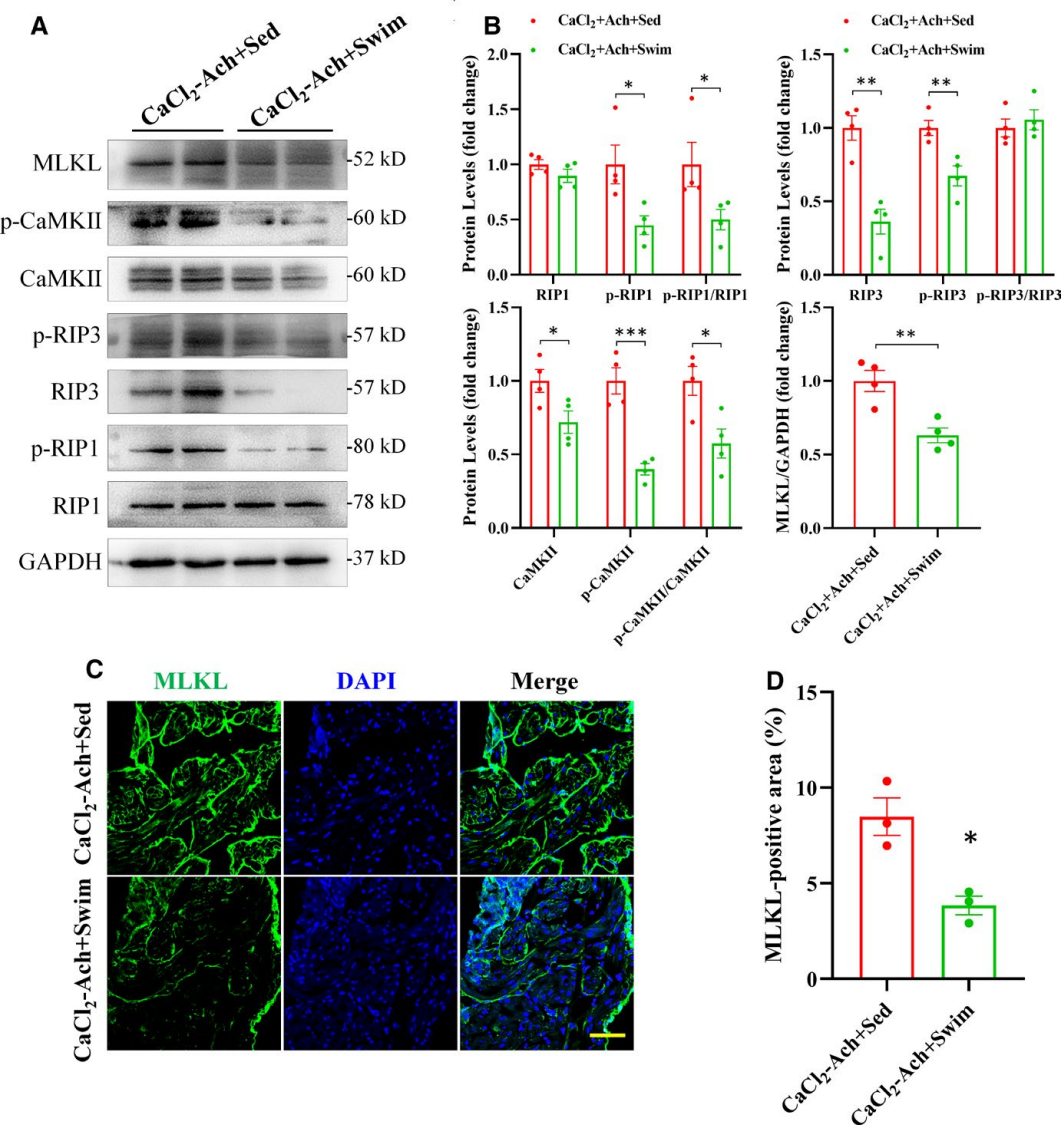
Swim exercise training inhibited cardiac necroptosis in CaCl_2_‐Ach‐induced AF mice. (A)–(B) Representative images and quantitative analysis showed significantly decreased level of RIP1, RIP3, CaMKII and MLKL or their phosphorylated forms in CaCl_2_‐Ach+Swim group (*n* = 4) compared with CaCl_2_‐Ach+Sed group (*n* = 4). (C)–(D) Representative images and quantitative analysis showed significantly decreased cell membrane translocation of MLKL in CaCl_2_‐Ach+Swim group (*n* = 3) compared with CaCl_2_‐Ach+Sed group (*n* = 3). Scale bar: 100 µm. ^*^ *p* < 0.05, ^**^ *p* < 0.01, ^***^ *p* < 0.001. CaCl_2_, calcium chloride; Ach, acetylcholine; AF, atrial fibrillation; Sed, sedentary

To further identify whether necroptosis was involved in swim exercise‐conferred benefits on AF, we evaluated necroptotic signaling in CaCl_2_‐Ach‐induced AF mice with or without 3‐week swim exercise training. As shown in Figure 6A,B, swim training decreased protein levels of RIP1, RIP3 or their phosphorylated forms in CaCl_2_‐Ach‐induced AF mice, indicating the inhibition of cardiac necroptosis by swim exercise training in CaCl_2_‐Ach‐induced AF mice.

Furthermore, after 3 weeks’ swim exercise training, protein levels of CaMKII and its phosphorylated form as well as expression of MLKL and its cell membrane translocation were significantly decreased in CaCl_2_‐Ach+Swim group compared with CaCl_2_‐Ach+Sed group (Figure 6). These findings highlighted that aerobic exercise may confer benefits on AF via inhibiting cardiac necroptosis.

## 4 DISCUSSION

Necroptosis plays a critical role in the development of cardiovascular diseases, but the role of necroptosis in AF and exercise‐mediated cardioprotection against AF is still unknown. In this study, we tend to explore (1) the role of necroptosis in AF pathogenesis and (2) whether 3‐week swim exercise training could attenuate AF and the role of necroptosis in this process. Our results showed cardiac necroptosis occurred in CaCl_2_‐Ach (or HFD)‐induced AF mice, and inhibiting necroptosis attenuated AF susceptibility and reversed atrial structural remodeling in both AF models. Furthermore, 3‐week swim exercise training could inhibit cardiac necroptosis, thus protecting the heart against CaCl_2_‐Ach‐induced AF.

Calcium overload has long been recognized as crucial for triggering AF because it may cause delayed after depolarizations/triggered activity as well as atrial structural remodeling. In line with this, Ach releasing can activate Ach‐sensitive potassium current (*I*_KACh_), consequently shortening action potential duration, increasing atria vulnerability and facilitating re‐entry formation, thus maintaining AF.^26^, ^27^ Early in 1982, Zhao and Guo,^28^ reported CaCl_2_‐Ach‐induced AF in mice. Later, more and more researchers used different dosage of CaCl_2_‐Ach mixture to establish an AF model in rats.^24^, ^29^, ^30^, ^31^, ^32^ Therefore, we used CaCl_2_ (10 mg/kg)‐Ach (66 µg/kg) mixture to establish a mouse AF model via tail vein injection and found increased AF susceptibility only after 3 weeks of CaCl_2_‐Ach administration, whereas no significant changes were observed earlier (Figure S2). Obesity has been linked to AF and recognized as an independent risk factor for AF recently.^33^ Obese patients showed cardiac hypertrophy, LA dilation, atrial fibrosis and inflammation,^33^ contributing to AF triggering and progression. Growing evidence proves HFD‐induced obesity also causes atrial inflammation and fibrosis, mediating atrial remodeling and AF in animal models.^34^, ^35^, ^36^, ^37^ Thus, we fed mice HFD containing 60 kcal% fat to mimic obesity and established an obesity‐induced mouse AF model. We found mice started to meet the standard of obesity at the end of week 9 (Figure 3B) and showed enhanced AF susceptibility at the end of week 12 (Figures 3C–E and S3).

Necroptosis is a regulated necrotic cell death emerging as a potential molecular target for cardiovascular diseases, especially age‐related heart disease.^8^ AF is an age‐related arrhythmia, and its prevalence increases with advancing age, rising from 0.7% in persons aged 55–59 years to 17.8% in those aged ≥85 years.^38^ Here we demonstrated that necroptosis was stimulated in CaCl_2_‐Ach‐induced AF mice (Figure 2), whereas inhibition of necroptosis via Nec‐1 attenuated CaCl_2_‐Ach‐induced AF burden and atrial structural remodeling (Figure 1), implicating the critical role of necroptosis in AF pathogenesis. In line with this, we found increased AF susceptibility and atrial fibrosis in HFD‐induced obesity mice and decreased AF susceptibility and atrial fibrosis after Nec‐1 administration (Figure 3), further supporting that necroptosis was required for AF pathogenesis independent of AF models. These are consistent with previous reports showing necroptosis and downstream targets play a crucial role in fibrosis, a hallmark of AF. Lee et al.^11^ revealed necroptosis was involved in the pathogenesis of lung fibrosis, and RIP3 knockout as well as RIP1 inhibition by Nec‐1 attenuated lung inflammation and fibrosis. Wei et al.^12^ proved RIP3 deficiency could mitigate liver fibrosis in mice. Moreover, CaMKII is also implicated to activate a set of downstream signaling to promote fibrosis such as TGFβ‐mediated collagen and fibronectin genes expression, whereas CaMKII silencing ameliorates TGFβ‐mediated fibrosis.^13^ To our knowledge, this is the first report of the link between necroptosis and AF pathogenesis, providing a new therapeutic target for prevention and treatment of AF.

Mechanistic link between necroptosis and atrial remodeling or AF pathophysiology has been widely explored. On one hand, compensatory proliferation of fibroblasts could be induced in response to loss of cardiomyocytes caused by necroptosis to maintain tissue homeostasis. On the other hand, necroptotic cells could release cellular contents, resulting in inflammatory^39^ and fibrotic responses,^40^ consequently leading to the development of myocardial fibrosis in severe cardiac pathological conditions. A growing body of evidence demonstrates the existence of inflammation and fibrosis in AF patients and experimental AF models such as CaCl_2_‐Ach‐induced AF and HFD‐induced AF.^30^, ^36^, ^41^ Therefore, necroptosis, and resultant activation of inflammation and fibrosis, might be important pathogenic factors of AF. Of note, up‐regulation of necroptosis, accompanied by chronic activation of inflammation and fibrosis, is commonly observed in the elderly,^42^ thus raising the possibility that necroptosis is considered as the potential mechanism of age‐related diseases such as AF, and providing a therapeutic target for AF prevention and management in the elderly. In addition to inflammation and fibrosis, metabolic syndrome, such as insulin resistance, is also widely implicated in AF pathogenesis. Polina et al.^43^ showed that loss of insulin signaling may contribute to AF in type 1 diabetes and Maria et al.^44^ showed that insulin treatment reduces susceptibility to AF in type 1 diabetic mice. In line with this, we did observe that both CaCl_2_‐Ach‐induced AF mice and HFD‐induced obese mice exhibited impaired glucose tolerance compared with control mice (data not shown). However, inhibition of necroptosis with Nec‐1 showed unchanged glucose tolerance in CaCl_2_‐Ach‐induced AF mice (data not shown), suggesting impaired insulin sensitivity might occur prior to necroptosis in AF pathogenesis, but not vice versa.

It is widely accepted that exercise is medicine for cardiovascular diseases. Hou et al.^45^ proved 4 weeks of high‐intensity swim training (twice daily, 90 min per session) protects against myocardial ischemia/reperfusion injury accompanied by mild cardiac hypertrophy in rats. Wang et al. revealed 3–9 weeks of moderate‐intensity swim training (5 days per week, progressively to 1 h daily) improved heart function and reduced cardiac hypertrophy and fibrosis in heart failure mice.^46^ However, in the context of AF, this relationship seems to be more complicated. Aschar‐Sobbi et al.^18^ found 6 weeks of high‐intensity swim training (twice daily, 90 min per session) increased AF susceptibility of mice, causing apparent left ventricle enlargement and hypertrophy. This is probably due to the exercise intensity, as high‐intensity exercise may promote myocardial hypertrophy, atrial dilation and atrial fibrosis,^17^ providing structural substrates for AF development. Therefore, in this study, we tend to explore whether moderate‐intensity swim exercise (60 min/day, once daily for 3 weeks) confers benefits on AF and found this exercise training protocol significantly reduced AF susceptibility and structural remodeling in CaCl_2_‐Ach‐induced AF mice, without causing apparent cardiac hypertrophy (Table S1). Our researches in this study and others support the notion that the effect of exercise on AF follows a J‐shape phenomenon,^47^ which means both high‐intensity exercise and sedentary lifestyle may raise the risk of AF whereas low to moderate exercise can conversely protect the heart against AF. These findings indicated AF patients should be more careful about exercise intensity, and low to moderate exercise intensity may be a preferred exercise modality for AF management without arrhythmogenic effects.

Increasing studies have demonstrated exercise was implicated to reduce cardiac apoptosis and necrosis.^48^ High‐intensity interval training exercise has previously been shown to prevent cardiac remodeling after myocardial ischemia/reperfusion injury via targeting necroptosis.^23^ In line with this, our results showed that 3‐week swim exercise training inhibited necroptotic signaling in CaCl_2_‐Ach‐induced AF mice. These results prompt the question as to how exercise inhibits necroptotic signaling. Previous researches have shown that oxidative stress and AMPK were upstream mechanisms that regulate necroptosis, and autophagy was linked to necroptosis,though we did not detect any significant change in cardiac oxidative stress between sedentary and exercised AF mice (data not shown), AMPK/mTOR‐mediated autophagy signaling was apparently enhanced in exercised mice compared with sedentary mice (Figure S4). Therefore, activated autophagy after swim exercise might lead to decreased necroptosis in AF mice. A further understanding of the molecular mechanisms can help to identify potential targets involved in exercise‐conferred cardioprotection and reveal novel therapeutic strategies for AF prevention and treatment.

There are some limitations in our research. First, we did not record spontaneous AF in mice. Second, gene intervention to enhance or reduce necroptosis was not performed to further illustrate the relationship between necroptosis and AF. Finally, we did not identify how exercise regulated necroptosis in the present study. Therefore, our research is still observational, and further studies are needed to clarify these issues.

## 5 CONCLUSION

In the present study, we investigated the role of necroptosis in AF and exercise‐conferred benefits on AF. First, our pharmacological studies demonstrated that necroptosis was attributable to AF pathogenesis. Next, improving physical activity via 3‐week swim training (60 min/day, once daily) reduced AF susceptibility in CaCl_2_‐Ach‐induced AF mice, mechanistically via suppressing necroptosis. Taken together, our findings identify necroptosis as a potential therapeutical target for AF treatment and highlight that aerobic exercise may confer benefits on AF via inhibiting cardiac necroptosis.

## CONFLICT OF INTEREST

The author declares that there is no conflict of interest that could be perceived as prejudicing the impartiality of the research reported.

## AUTHOR CONTRIBUTIONS

**Yuping Fu:** Conceptualization (lead); Data curation (lead); Formal analysis (lead); Writing‐original draft (lead). **Tiannan Jiang:** Data curation (equal); Formal analysis (equal); Methodology (lead); Writing‐original draft (supporting). **Hongke Sun:** Data curation (supporting); Methodology (equal). **Tong Li:** Formal analysis (supporting); Methodology (equal). **Feng Gao:** Formal analysis (supporting); Methodology (supporting). **Boyuan Fan:** Methodology (supporting). **Xiaoli Li:** Methodology (supporting). **Xinghua Qin:** Conceptualization (equal); Funding acquisition (equal); Writing‐review & editing (equal). **Qiangsun Zheng:** Conceptualization (equal); Funding acquisition (equal); Supervision (equal); Writing‐review & editing (equal).

## Supporting information

Figures S1–S4

Table S1

## Data Availability

The data that support the findings of this study are available from the corresponding author upon reasonable request.

## References

[1] Andrade J, Khairy P, Dobrev D, Nattel S. The clinical profile and pathophysiology of atrial fibrillation: relationships among clinical features, epidemiology, and mechanisms. Circ Res. 2014;114:1453‐1468. 24763464 10.1161/CIRCRESAHA.114.303211

[2] Nattel S, Harada M. Atrial remodeling and atrial fibrillation: recent advances and translational perspectives. J Am Coll Cardiol. 2014;63:2335‐2345. 24613319 10.1016/j.jacc.2014.02.555

[3] Konstantinidis K, Whelan RS, Kitsis RN. Mechanisms of cell death in heart disease. Arterioscler Thromb Vasc Biol. 2012;32:1552‐1562. 22596221 10.1161/ATVBAHA.111.224915 PMC3835661

[4] Trappe K, Thomas D, Bikou O, et al. Suppression of persistent atrial fibrillation by genetic knockdown of caspase 3: a pre‐clinical pilot study. Eur Heart J. 2013;34:147‐157. 21785105 10.1093/eurheartj/ehr269

[5] Xu GJ, Gan TY, Tang BP, et al. Accelerated fibrosis and apoptosis with ageing and in atrial fibrillation: Adaptive responses with maladaptive consequences. Exp Ther Med. 2013;5:723‐729. 23403858 10.3892/etm.2013.899 PMC3570166

[6] Holler N, Zaru R, Micheau O, et al. Fas triggers an alternative, caspase‐8‐independent cell death pathway using the kinase RIP as effector molecule. Nat Immunol. 2000;1:489‐495. 11101870 10.1038/82732

[7] Gupta K, Phan N, Wang Q, Liu B. Necroptosis in cardiovascular disease ‐ a new therapeutic target. J Mol Cell Cardiol. 2018;118:26‐35. 29524460 10.1016/j.yjmcc.2018.03.003 PMC5940532

[8] Li C, Mu N, Gu C, et al. Metformin mediates cardioprotection against aging‐induced ischemic necroptosis. Aging Cell. 2020;19:e13096. 31944526 10.1111/acel.13096 PMC6996959

[9] Linkermann A, Green DR. Necroptosis. N Engl J Med. 2014;370:455‐465. 24476434 10.1056/NEJMra1310050 PMC4035222

[10] Zhang T, Zhang Y, Cui M, et al. CaMKII is a RIP3 substrate mediating ischemia‐ and oxidative stress‐induced myocardial necroptosis. Nat Med. 2016;22:175‐182. 26726877 10.1038/nm.4017

[11] Lee JM, Yoshida M, Kim MS, et al. Involvement of alveolar epithelial cell necroptosis in idiopathic pulmonary fibrosis pathogenesis. Am J Respir Cell Mol Biol. 2018;59:215‐224. 29444413 10.1165/rcmb.2017-0034OC

[12] Wei S, Zhou H, Wang Q, et al. RIP3 deficiency alleviates liver fibrosis by inhibiting ROCK1‐TLR4‐NF‐kappaB pathway in macrophages. FASEB J. 2019;33:11180‐11193. 31295018 10.1096/fj.201900752R

[13] Mukherjee S, Sheng W, Sun R, Janssen LJ. Ca(2+)/calmodulin‐dependent protein kinase IIbeta and IIdelta mediate TGFbeta‐induced transduction of fibronectin and collagen in human pulmonary fibroblasts. Am J Physiol Lung Cell Mol Physiol. 2017;312:L510‐L519. 28130256 10.1152/ajplung.00084.2016

[14] Iwasaki YK, Nishida K, Kato T, Nattel S. Atrial fibrillation pathophysiology: implications for management. Circulation. 2011;124:2264‐2274. 22083148 10.1161/CIRCULATIONAHA.111.019893

[15] Pedersen BK, Saltin B. Exercise as medicine ‐ evidence for prescribing exercise as therapy in 26 different chronic diseases. Scand J Med Sci Sports. 2015;25(Suppl 3):1‐72. 10.1111/sms.12581 26606383

[16] Elliott AD, Mahajan R, Pathak RK, Lau DH, Sanders P. Exercise training and atrial fibrillation: further evidence for the importance of lifestyle change. Circulation. 2016;133:457‐459. 26733608 10.1161/CIRCULATIONAHA.115.020800

[17] Guasch E, Benito B, Qi X, et al. Atrial fibrillation promotion by endurance exercise: demonstration and mechanistic exploration in an animal model. J Am Coll Cardiol. 2013;62:68‐77. 23583240 10.1016/j.jacc.2013.01.091

[18] Aschar‐Sobbi R, Izaddoustdar F, Korogyi AS, et al. Increased atrial arrhythmia susceptibility induced by intense endurance exercise in mice requires TNFalpha. Nat Commun. 2015;6:6018. 25598495 10.1038/ncomms7018 PMC4661059

[19] Mont L, Elosua R, Brugada J. Endurance sport practice as a risk factor for atrial fibrillation and atrial flutter. Europace. 2009;11:11‐17. 18988654 10.1093/europace/eun289 PMC2638655

[20] Morseth B, Graff‐Iversen S, Jacobsen BK, et al. Physical activity, resting heart rate, and atrial fibrillation: the Tromsø Study. Eur Heart J. 2016;37(29):2307‐2313. 26966149 10.1093/eurheartj/ehw059 PMC4986028

[21] Mozaffarian D, Furberg CD, Psaty BM, Siscovick D. Physical activity and incidence of atrial fibrillation in older adults: the cardiovascular health study. Circulation. 2008;118(8):800‐807. 18678768 10.1161/CIRCULATIONAHA.108.785626 PMC3133958

[22] Hegbom F, Stavem K, Sire S, Heldal M, Orning OM, Gjesdal K. Effects of short‐term exercise training on symptoms and quality of life in patients with chronic atrial fibrillation. Int J Cardiol. 2007;116:86‐92. 16815571 10.1016/j.ijcard.2006.03.034

[23] Ghardashi Afousi A, Gaeini A, Rakhshan K, Naderi N, Darbandi Azar A, Aboutaleb N. Targeting necroptotic cell death pathway by high‐intensity interval training (HIIT) decreases development of post‐ischemic adverse remodelling after myocardial ischemia / reperfusion injury. J Cell Commun Signal. 2019;13:255‐267. 30073629 10.1007/s12079-018-0481-3 PMC6498245

[24] Chen C, Gong T, Tang Y, et al. Establishment of artial fibrillation model in SD rats. Lab Anim Sci. 2009;26:1‐4.

[25] Fan B, Wang H, Wu T, et al. Electrophysiological measurement of rat atrial epicardium using a novel stereotaxic apparatus. Int Heart J. 2019;60:400‐410. 30799380 10.1536/ihj.18-215

[26] Chen PS, Chen LS, Fishbein MC, Lin SF, Nattel S. Role of the autonomic nervous system in atrial fibrillation: pathophysiology and therapy. Circ Res. 2014;114:1500‐1515. 24763467 10.1161/CIRCRESAHA.114.303772 PMC4043633

[27] Linz D, Ukena C, Mahfoud F, Neuberger HR, Bohm M. Atrial autonomic innervation: a target for interventional antiarrhythmic therapy? J Am Coll Cardiol. 2014;63:215‐224. 24140663 10.1016/j.jacc.2013.09.020

[28] Zhao ZY, Guo YM. CaCl_2_‐ACh induced atrial fibrillation (flutter) in mice. Zhongguo Yao Li Xue Bao. 1982;3:185‐188. 6216728

[29] Zou D, Geng N, Chen Y, et al. Ranolazine improves oxidative stress and mitochondrial function in the atrium of acetylcholine‐CaCl_2_ induced atrial fibrillation rats. Life Sci. 2016;156:7‐14. 27208652 10.1016/j.lfs.2016.05.026

[30] Yang Q, Wu G, Han L, et al. Taurine reverses atrial structural remodeling in Ach‐CaCl_2_ induced atrial fibrillation rats. Adv Exp Med Biol. 2017;975(Pt 2):831‐841. 28849503 10.1007/978-94-024-1079-2_65

[31] Li Y, Song B, Xu C. Effects of Guanfu total base on Bcl‐2 and Bax expression and correlation with atrial fibrillation. Hellenic J Cardiol. 2018;59:274‐278. 29501704 10.1016/j.hjc.2018.02.009

[32] Zhou Q, Chen B, Chen X, et al. Arnebiae Radix prevents atrial fibrillation in rats by ameliorating atrial remodeling and cardiac function. J Ethnopharmacol. 2020;248:112317. 31629862 10.1016/j.jep.2019.112317

[33] Lavie CJ, Pandey A, Lau DH, Alpert MA, Sanders P. Obesity and atrial fibrillation prevalence, pathogenesis, and prognosis: effects of weight loss and exercise. J Am Coll Cardiol. 2017;70:2022‐2035. 29025560 10.1016/j.jacc.2017.09.002

[34] Takahashi K, Sasano T, Sugiyama K, et al. High‐fat diet increases vulnerability to atrial arrhythmia by conduction disturbance via miR‐27b. J Mol Cell Cardiol. 2016;90:38‐46. 26654778 10.1016/j.yjmcc.2015.11.034

[35] Meng T, Cheng G, Wei Y, et al. Exposure to a chronic high‐fat diet promotes atrial structure and gap junction remodeling in rats. Int J Mol Med. 2017;40:217‐225. 28498436 10.3892/ijmm.2017.2982

[36] Kondo H, Abe I, Gotoh K, et al. Interleukin 10 treatment ameliorates high‐fat diet‐induced inflammatory atrial remodeling and fibrillation. Circ Arrhythm Electrophysiol. 2018;11:e006040. 29748196 10.1161/CIRCEP.117.006040

[37] Zhang Y, Yang S, Fu J, Liu A, Liu D, Cao S. Inhibition of endoplasmic reticulum stress prevents high‐fat diet mediated atrial fibrosis and fibrillation. J Cell Mol Med. 2020;24:13660‐13668. 33135380 10.1111/jcmm.15816 PMC7754029

[38] Heeringa J, van der Kuip DA, Hofman A, et al. Prevalence, incidence and lifetime risk of atrial fibrillation: the Rotterdam study. Eur Heart J. 2006;27:949‐953. 16527828 10.1093/eurheartj/ehi825

[39] Pasparakis M, Vandenabeele P. Necroptosis and its role in inflammation. Nature. 2015;517:311‐320. 25592536 10.1038/nature14191

[40] Mack M. Inflammation and fibrosis. Matrix Biol. 2018;68–69:106‐121. 10.1016/j.matbio.2017.11.010 29196207

[41] Hu YF, Chen YJ, Lin YJ, Chen SA. Inflammation and the pathogenesis of atrial fibrillation. Nat Rev Cardiol. 2015;12:230‐243. 25622848 10.1038/nrcardio.2015.2

[42] Deepa SS, Unnikrishnan A, Matyi S, Hadad N, Richardson A. Necroptosis increases with age and is reduced by dietary restriction. Aging Cell. 2018;17:e12770. 29696779 10.1111/acel.12770 PMC6052392

[43] Polina I, Jansen HJ, Li T, et al. Loss of insulin signaling may contribute to atrial fibrillation and atrial electrical remodeling in type 1 diabetes. Proc Natl Acad Sci USA. 2020;117:7990‐8000. 32198206 10.1073/pnas.1914853117 PMC7148583

[44] Maria Z, Campolo AR, Scherlag BJ, Ritchey JW, Lacombe VA. Insulin treatment reduces susceptibility to atrial fibrillation in Type 1 diabetic mice. Front Cardiovasc Med. 2020;7:134. 32903422 10.3389/fcvm.2020.00134 PMC7434932

[45] Hou Z, Qin X, Hu Y, et al. Longterm exercise‐derived exosomal miR‐342‐5p: a novel exerkine for cardioprotection. Circ Res. 2019;124:1386‐1400. 30879399 10.1161/CIRCRESAHA.118.314635

[46] Wang B, Xu M, Li W, et al. Aerobic exercise protects against pressure overload‐induced cardiac dysfunction and hypertrophy via β3‐AR‐nNOS‐NO activation. PLoS One. 2017;12:e0179648. 28622359 10.1371/journal.pone.0179648 PMC5473571

[47] Morseth B, Lochen ML, Ariansen I, Myrstad M, Thelle DS. The ambiguity of physical activity, exercise and atrial fibrillation. Eur J Prev Cardiol. 2018;25:624‐636. 29411631 10.1177/2047487318754930

[48] French JP, Hamilton KL, Quindry JC, Lee Y, Upchurch PA, Powers SK. Exercise‐induced protection against myocardial apoptosis and necrosis: MnSOD, calcium‐handling proteins, and calpain. FASEB J. 2008;22:2862‐2871. 18417547 10.1096/fj.07-102541 PMC2493460

