# Supplementary material for: Necroptosis is required for atrial fibrillation and involved in aerobic exercise‐conferred cardioprotection
Source: J Cell Mol Med. 2021 Jul 20;25(17):8363–75. doi: 10.1111/jcmm.16796 (PMC8419184; doi:10.1111/jcmm.16796)
Supplement: Supplementary file 1 — Figures S1–S4 [file JCMM-25-8363-s002.docx]

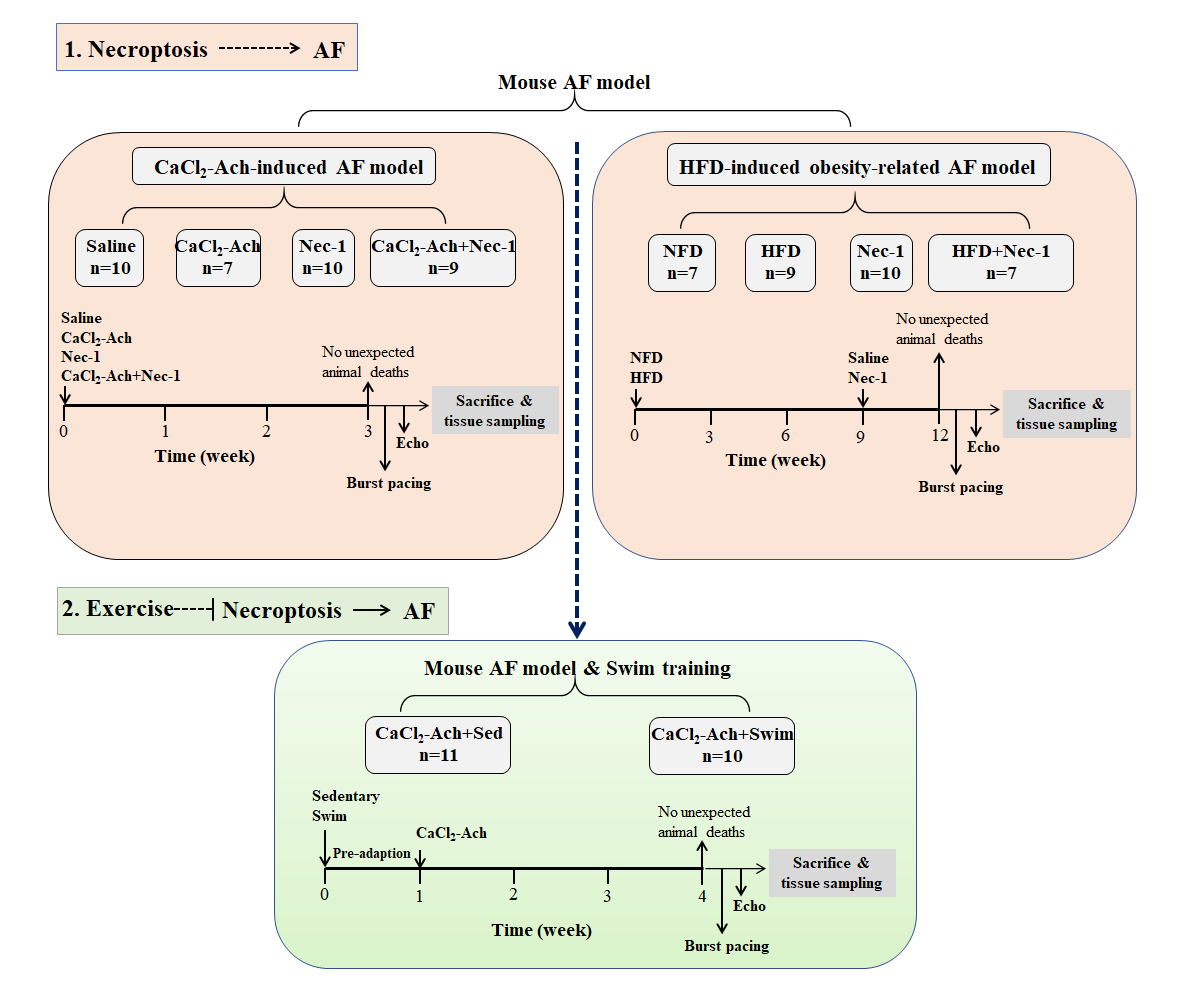


**Figure S1. Experimental framework.** AF, atrial fibrillation; CaCl_2_, calcium chloride; Ach, acetylcholine; Nec-1, necrostatin-1; Echo, echocardiography; NFD, normal-fat diet; HFD, high-fat diet; Sed, sedentary.


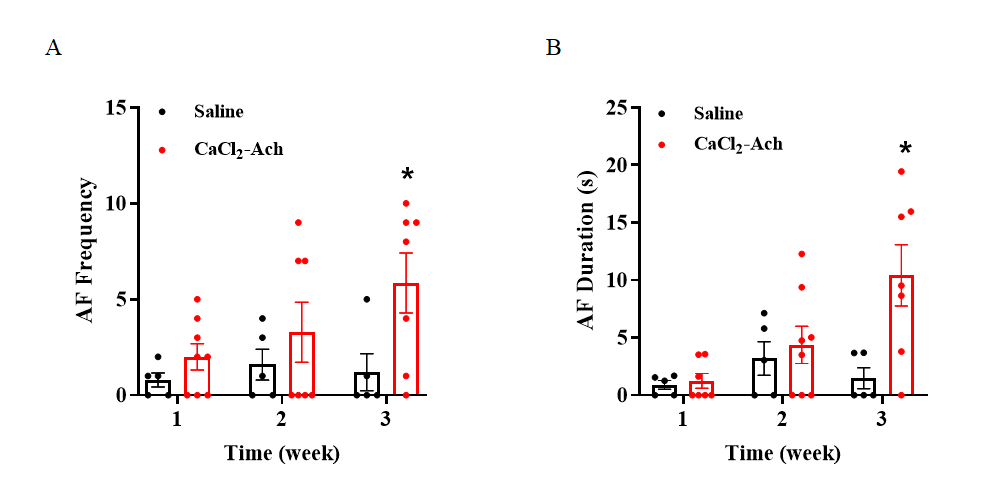


**Figure S2.** **AF susceptibility after CaCl_2_-Ach administration in week 1, 2, and 3.** (A) Bar graphs showed increased AF frequency after 3-week CaCl_2_-Ach administration while no statistical differences in week 1 and 2. (B) Bar graphs showed increased AF duration after 3-week CaCl_2_-Ach administration while no statistical differences in week 1 and 2. n = 5 in Saline group, n=7 in CaCl_2_-Ach group, **P* < 0.05. CaCl_2_, calcium chloride; Ach, acetylcholine.


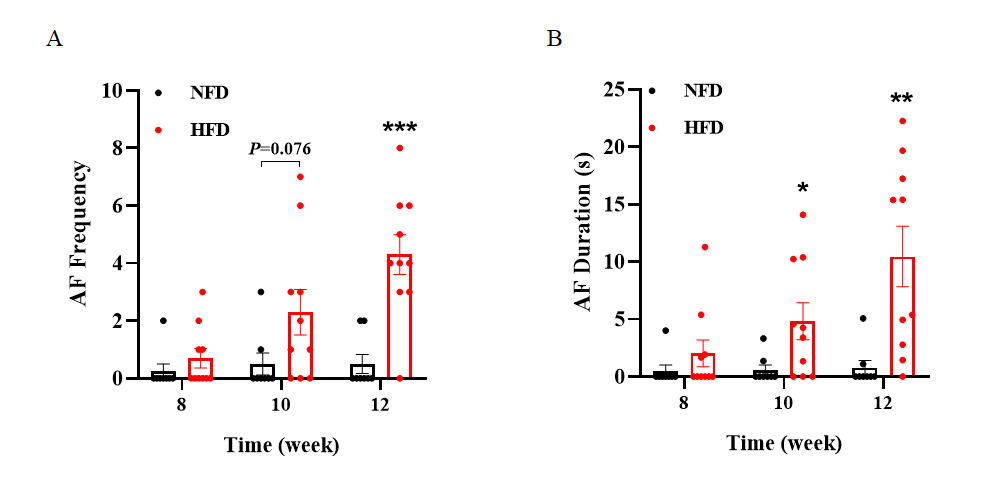


**Figure S3. AF susceptibility after HFD feeding in week 8, 10 and 12.** (A) Bar graphs showed increased AF frequency after 12-week HFD feeding while no statistical differences in week 8 and 10. (B) Bar graphs showed increased AF duration after 10 and 12-week HFD feeding while no statistical differences in week 8. n = 8 in NFD group, n=10 in HFD group, **P* < 0.05, ***P* < 0.01, ****P* < 0.001. NFD, normal-fat diet; HFD, high-fat diet.


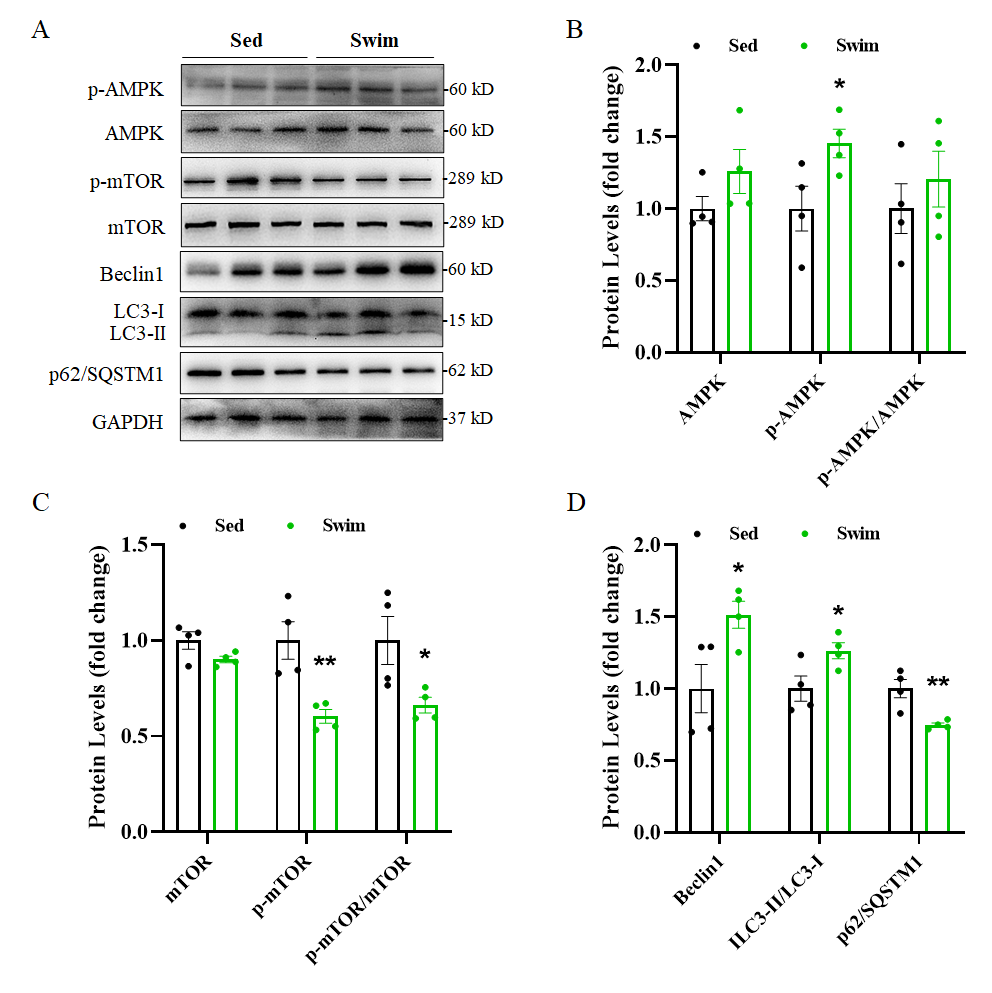


**Figure S4. Swim training increased AMPK/mTOR-mediated atrial autophagy.** (A) Representative images of protein expression of AMPK/mTOR-mediated autophagy signaling. (B-D) Quantitative analysis showed significantly increased p-AMPK, Beclin1, LC3-II/LC3-I levels and decreased p-mTOR and p62/SQSTM1 levels in Swim group (n = 4) compared with Sed group (n = 4). **P* < 0.05, ***P* < 0.01. Sed, sedentary.
