# Supplementary material for: Necroptosis is required for atrial fibrillation and involved in aerobic exercise‐conferred cardioprotection
Source: J Cell Mol Med. 2021 Jul 20;25(17):8363–75. doi: 10.1111/jcmm.16796 (PMC8419184; doi:10.1111/jcmm.16796)
Supplement: Supplementary file 2 — Table S1 [file JCMM-25-8363-s001.docx]

**Supplementary Material**

**Table S1. Basal echocardiographic parameters**

| **Parameter** |  | **Group 1** | | | | **Group 3** | |
| --- | --- | --- | --- | --- | --- | --- | --- |
|  | **Saline**  (n = 4) | **CaCl_2_-Ach**  (n = 7) | **Nec-1**  (n = 7) | **CaCl_2_-Ach+Nec-1**  (n = 9) | **CaCl_2_-Ach+Sed**  (n = 10) | | **CaCl_2_-Ach+Swim**  (n = 7) |
| BW (g) | 21.23 ± 0.536 | 22.60 ± 0.265 | 23.43 ± 0.376 | 24.14 ± 0.452 | 22.32 ± 0.401 | | 20.46 ± 0.291 |
| HR (bpm) | 335.4 ± 8.807 | 286.9 ± 29.40 | 305.1 ± 5.313 | 306.3 ± 18.57 | 277.7 ± 10.23 | | 319.5 ± 10.02**^#^** |
| LV/BW (g/kg) | 3.344 ± 0.108 | 2.722 ± 0.095 | 2.975 ± 0.120 | 2.759 ± 0.141 | 2.969 ± 0.138 | | 2.979 ± 0.148 |
| **Structure** |  |  |  |  |  | |  |
| AO diameter (mm) | 1.689 ± 0.023 | 1.717 ± 0.054 | 1.670 ± 0.023 | 1.828 ± 0.037 | 1.694 ± 0.021 | | 1.774 ± 0.027**^#^** |
| LA diameter (mm) | 1.755 ± 0.052 | 2.152 ± 0.070****** | 1.852 ± 0.051 | 1.850 ± 0.066***** | 2.202 ± 0.089 | | 1.823 ± 0.051**^##^** |
| IVSd (mm) | 0.836 ± 0.055 | 0.789 ± 0.045 | 0.857 ± 0.034 | 0.835 ± 0.050 | 0.770 ± 0.028 | | 0.776 ± 0.031 |
| LVIDd (mm) | 3.592 ± 0.034 | 3.211 ± 0.115 | 3.455 ± 0.026 | 3.303 ± 0.169 | 3.457 ± 0.059 | | 3.405 ± 0.048 |
| LVPWd (mm) | 0.654 ± 0.044 | 0.745 ± 0.050 | 0.784 ± 0.031 | 0.736 ± 0.051 | 0.695 ± 0.035 | | 0.634 ± 0.030 |
| IVSs (mm) | 1.192 ± 0.107 | 1.197 ± 0.072 | 1.222 ± 0.032 | 1.299 ± 0.063 | 1.164 ± 0.033 | | 1.159 ± 0.045 |
| LVIDs (mm) | 2.384 ± 0.090 | 2.042 ± 0.174 | 2.384 ± 0.060 | 2.009 ± 0.185 | 2.298 ± 0.077 | | 2.299 ± 0.076 |
| LVPWs (mm) | 1.159 ± 0.111 | 1.231 ± 0.121 | 1.161 ± 0.047 | 1.280 ± 0.073 | 1.109 ± 0.053 | | 1.017 ± 0.076 |
| **Function** |  |  |  |  |  | |  |
| EF (%) | 62.91 ± 3.779 | 67.39 ± 4.315 | 62.86 ± 2.046 | 69.19 ± 3.603 | 63.09 ± 2.033 | | 61.47 ± 3.122 |
| FS (%) | 33.49 ± 2.612 | 36.92 ± 3.275 | 32.46 ± 1.273 | 38.91 ± 3.045 | 33.62 ± 1.626 | | 32.45 ± 2.296 |

Values were expressed as mean ± SEM; **^*^***P* < 0.05 vs. CaCl_2_-Ach group, **^**^***P* < 0.01 vs. Saline group, **^#^***P* < 0.05 vs. CaCl_2_-Ach+Sed group, **^##^***P* < 0.01 vs. CaCl_2_-Ach+Sed group; BW, body weight; HR, heart rate; LV, left ventricle; AO, aortic root; LA, left atrium; IVSd, end-diastolic interventricular septum thickness; LVIDd, end-diastolic LV internal dimension; LVPWd, end-diastolic LV posterior wall thickness; IVSs, end- systolic interventricular septum thickness; LVIDs, end-systolic LV internal dimension; LVPWs, end-systolic LV posterior wall thickness; EF, ejection fraction; FS, fraction shortening.
